# Development of a ferromagnetic component in the superconducting state of Fe-excess Fe_1.12_Te_1-x_Se_x_ by electronic charge redistribution

**DOI:** 10.1038/srep10951

**Published:** 2015-06-16

**Authors:** Wen-Hsien Li, Sunil K. Karna, Han Hsu, Chi-Yen Li, Chi-Hung Lee, Raman Sankar, Fang Cheng Chou

**Affiliations:** 1 Department of Physics, National Central University, Jhongli 32001, Taiwan; 2 Center for Condensed Matter Sciences, National Taiwan University, Taipei 10617, Taiwan

## Abstract

The general picture established so far for the links between superconductivity and magnetic ordering in iron chalcogenide Fe_1+y_(Te_1-x_Se_x_) is that the substitution of Se for Te directly drives the system from the antiferromagnetic end into the superconducting regime. Here, we report on the observation of a ferromagnetic component that developed together with the superconducting transition in Fe-excess Fe_1.12_Te_1-x_Se_x_ crystals using neutron and x-ray diffractions, resistivity, magnetic susceptibility and magnetization measurements. The superconducting transition is accompanied by a negative thermal expansion of the crystalline unit cell and an electronic charge redistribution, where a small portion of the electronic charge flows from around the Fe sites toward the Te/Se sites. First-principles calculations show consistent results, revealing that the excess Fe ions play a more significant role in affecting the magnetic property in the superconducting state than in the normal state and the occurrence of an electronic charge redistribution through the superconducting transition.

Superconductivity in α-FeSe has received special attention due to its simple crystalline structure^1^,^2^,^3^,^4^,^5^,^6^,^7^. It is known that the wavy layered α-FeSe structure can accommodate a significant amount of extra Fe ions^8^,^9^. These non-stoichiometric Fe ions (marked Fe(2)) occupy the interstitial sites that are slightly below the center positions of the Se square sublattice. The stoichiometric Fe ions in the FeSe layers are hereafter marked Fe(1). The development of superconductivity through Se-doping is mainly associated with alternation of the band structure and electron density at the Fermi level, since Te^2-^ and Se^2-^ have the same valence which will not directly cause significant differences in carrier doping, but the ionic radius of Se^2-^ is 14% smaller which can noticeably alter the band structure. A long-range antiferromagnetic (AFM) order with an incommensurate (ICM) wave vector of q = (0.346, 0, 0.5) has been reported^8^ in Fe_1.165_Te. The ICM wave vector changes to q = (0.38, 0, 0.5) in Fe_1.141_Te^8^. The system locks into a commensurate (CM) AFM structure in the compounds with a lower amount of excess Fe ions, where collinear AFM orders with a wave vector of q = (0.5, 0, 0.5) are seen in Fe_1.125_Te^9^ and Fe_1.068_Te^10^. These observations show that the magnetic propagation vector of Fe_1+y_Te can be tuned by adjusting the amount of excess Fe ions. Interestingly, short-range magnetic correlation survives in the superconducting regime^8^,^11^,^12^,^13^,^14^,^15^, where the interplay between the spin and charge degrees of freedom is clearly evident. More importantly, ferromagnetic ordering has been observed^16^ in the high temperature phase of FeTe_0.9_, which evolves into a ferrimagnetic structure below 63 K. There is indeed a net ferromagnetic component in the low temperature ferrimagnetic phase, suggesting that ferromagnetic coupling does exist in this class of materials. It is clear that the magnetic structure^12^,^13^,^17^ of the Fe-excess Fe_1+y_(Te_1-x_Se_x_) is fundamentally different from that of the stoichiometric Fe(Te_1-x_Se_x_). In addition, the excess Fe ions will alter the overall electronic configuration of the conduction and magnetic components. The goal of this study is to search for the ferromagnetic component that links directly to the superconductivity in Fe-excess Fe(Te,Se). Two magnetic phases are identified. The low-temperature magnetic phase, which coexists with superconductivity, involves the ordering of the Fe(1) and Fe(2) ions. The formation of superconducting pairs drives the electrons to flow from the Fe sites to the Te/Se sites, which alters the numbers of Fe^2+^ ions on the lattice and the excess sites. It is this change in valence of the magnetic sites that drives the system to develop an additional ferromagnetic component in the superconducting state.

## Results and discussion

Four sets of samples with nominal compositions of Fe_1.12_Te_1-x_Se_x_ (x = 0.4 and 0.6) and Fe_1+y_Te_0.6_Se_0.4_ (y = 0.03 and 0.08) were synthesized using the solid-state reaction technique. The process resulted in the formation of many crystals in each batch. A number of small crystals from each batch were crushed into powdered form for powder diffraction measurement. The chemical compositions obtained from high-resolution neutron powder diffraction (collected on the high-resolution powder diffractometer Echidna at ANSTO) together with energy dispersive x-ray spectroscopy were Fe_1.12_Te_0.61_Se_0.39_ for the x = 0.4, Fe_1.1_Te_0.39_Se_0.59_ for the x = 0.6, Fe_1.03_Te_0.61_Se_0.39_ for the y = 0.03, and Fe_1.08_Te_0.61_Se_0.39_ for the y 0.08 samples. Details of the sample fabrication and characterization can be found in the supplementary material. A single crystal of Fe_1.12_Te_0.6_Se_0.4_ used in the present measurements weighed 115 mg, with a size of 8 × 4 × 2 mm^3^.

**Figure 1:**
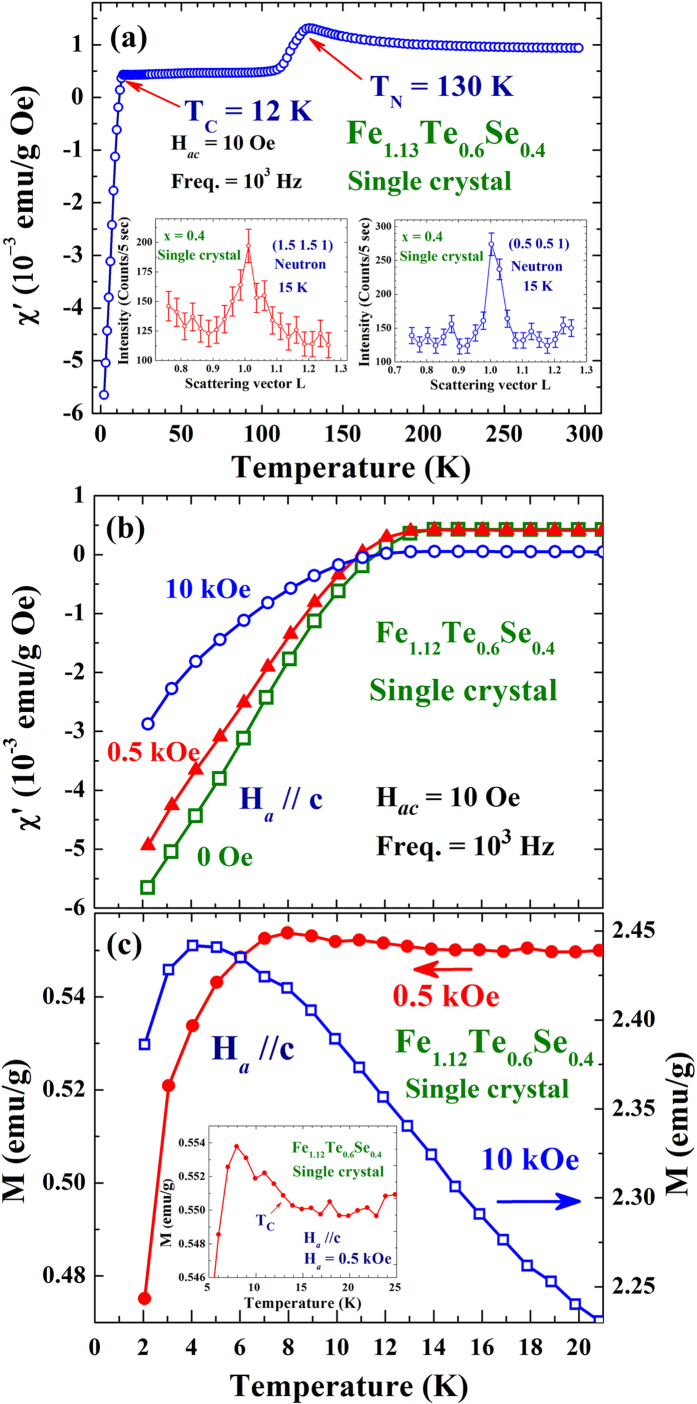
(**a**) χ′(T) curve of the x = 0.4 crystal, measured using a probing field with a root-mean-square strength of 10 Oe and a frequency of 10^3^ Hz. Two anomalies at 130 and 12 K are clearly evident. The insets show the (1/2 1/2 1) and (3/2 3/2 1) reflections obtained at 15 K. (**b**) χ′(T) curves of the x = 0.4 crystal, measured at H_*a *_= 0 (open squares), 0.5 kOe (filled triangles) and 10 kOe (open circles). (**c**) M(T) curves of the x = 0.4 crystal, measured at H_*a*_ = 0.5 kOe (filled circles) and 10 kOe (open squares). The inset shows the M(T) curve in the critical regime using an expanded scale.

Two anomalies are clearly revealed in the temperature dependence of the in-phase component χ′ of the ac magnetic susceptibility of the x = 0.4 crystal (Fig. 1a). The one marked T_N_ = 130 K indicates the development of antiferromagnetic (AFM) ordering of the Fe ions, with a magnetic propagation vector of (1/2 1/2 1) (collected on the triple-axis spectrometer Taipan at ANSTO) (insets to Fig. 1a). The one marked T_C_ = 12 K, on the other hand, indicates the development of superconductivity. T_C_ and the amount of superconducting diamagnetic screening (SDS) are only slightly affected by the applied magnetic field H_*a*_ of 10 kOe (Fig. 1b). SDS is also evident in the temperature dependence of the magnetization M taken at H_*a*_ = 0.5 kOe, with positive values of M being obtained at all temperatures studied (Fig. 1c). Small but visible increases of M are seen on cooling to below T_C_ before SDS becomes dominating (inset to Fig. 1c). Increasing H_*a*_ to 10 kOe results in *a* significant increase of the values of M (~5 times larger), but the amount of SDS of M is only slightly affected. The χ′(T) and M(T) curves with a temperature range covering 2 to 300 K can be found in the supplementary material.

**Figure 2:**
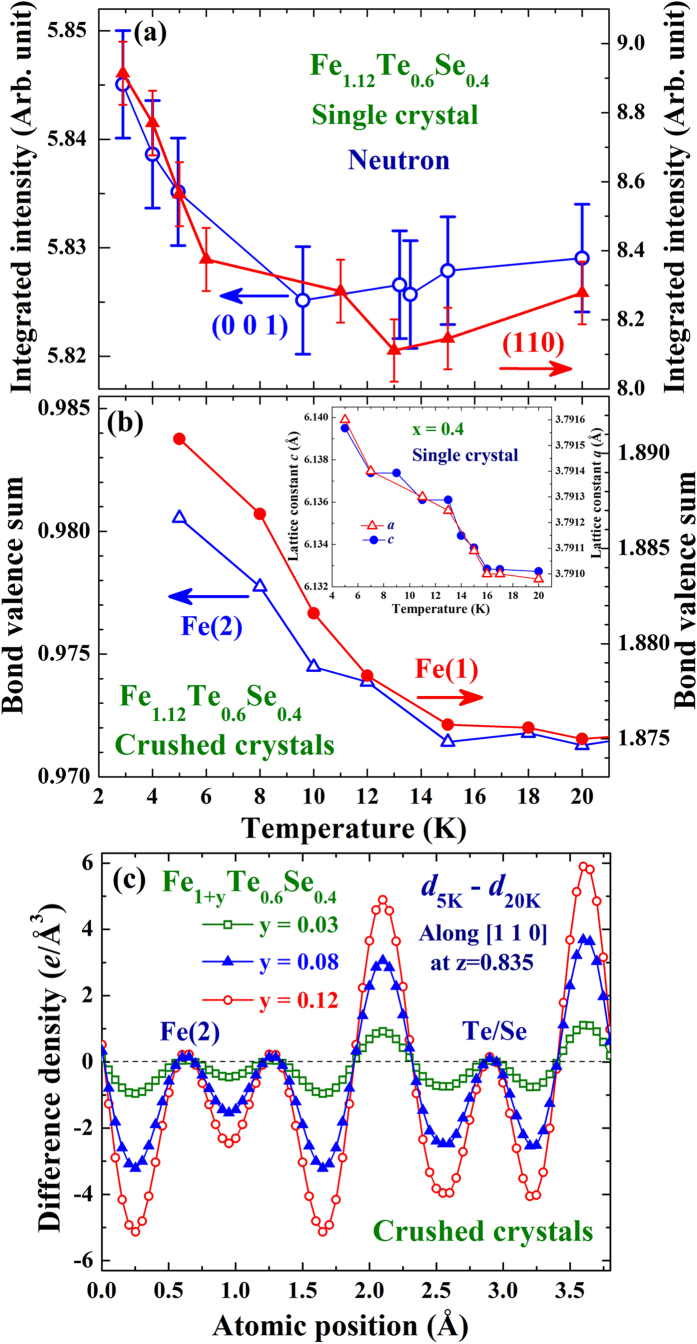
(**a**) Temperature dependencies of the integrated intensities of the (001) (open circles) and (110) (filled triangles) reflections of the x = 0.4 crystal. (**b**) Temperature dependence of the bond valence sum of the lattice iron Fe(1) (filled circles) and the excess iron Fe(2) (open triangles) of the x = 0.4 sample. The inset shows the temperature dependence of the lattice constants *a* and *c*. (**c**) Changes of the electronic charge density along the [110] crystallographic direction in the (0 0 0.835) plane upon cooling from 20 to 5 K of the x = 0.4 compounds with 3% (open squares), 10% (filled triangles) and 12% (open circles) excess Fe(2) densities.

Interestingly, a ferromagnetic (FM) component develops together with the superconductivity, where additional FM intensities, represented by the (001) and (110) intensities, appear in the neutron diffraction patterns upon cooling through the superconducting transition (Fig. 2a). Note that the AFM component, represented by the (1/2 1/2 1) intensity, remains unaltered through the superconducting transition. There is no noticeable change in the x-ray diffraction intensities of the (001) and (110) reflections and no structural change (via high-resolution neutron powder diffraction patterns) that can be identified upon cooling through the superconducting transition. The increases of the (001) and (110) intensities in neutron diffraction are hence mainly magnetic in origin. In addition, the increases in M upon cooling through the superconducting transition (inset to Fig. 1c) indicate that the FM moment developed in the high temperature regime of the transition is larger than the SDS could screen. However, the amount of increase in M (by 0.003 emu/g) on cooling from 12 to 8 K is beyond the detection of neutron diffraction at current sensitivity.

Upon cooling through the superconducting transition, the lattice constant along the crystallographic *c*-axis direction expands significantly by ~0.11%, while the in-plan lattice constant expands by only ~0.017% (inset to Fig. 2b). The chemical unit cell largely elongates along the *c*-axis direction on cooling through the superconducting transition. It appears that the neighboring Fe(Te/Se) layers are less connected in the superconducting state than in the normal state. Remarkably, the superconducting transition is accompanied by an electronic charge redistribution. The valences of Fe(1) and Fe(2) increase significantly upon cooling through the superconducting transition (Fig. 2b), showing that cooling causes a flow of electronic charges from the magnetic Fe(1) and Fe(2) sites onto the Te/Se sites. The changes in the electronic charge density along the representative [110] crystallographic direction in the (0 0 0.835) plane of the x = 0.4 compounds with 3%, 8% and 12% excess Fe(2) densities upon cooling from 20 to 5 K are illustrated in Fig. 2c. Those atomic positions with negative values represent the locations where electronic charges have flowed to the positions with positive values. Charge redistribution is also evident in the compounds with a lower Fe(2) density, but the effect is considerably reduced. These electron density maps were obtained by employing the GSAS program, starting with a profile refining the x-ray diffraction pattern, followed by calculation of the inverse Fourier transforms of the structure factors to extract the electron density distribution. The electron density contour map of a specific plane was then obtained by slicing the electron density in the vicinity, including 0.025 Å below and above the plane. The electronic charge density along a specific crystallographic direction was then obtained by cutting the density map along the selective direction.

**Figure 3:**
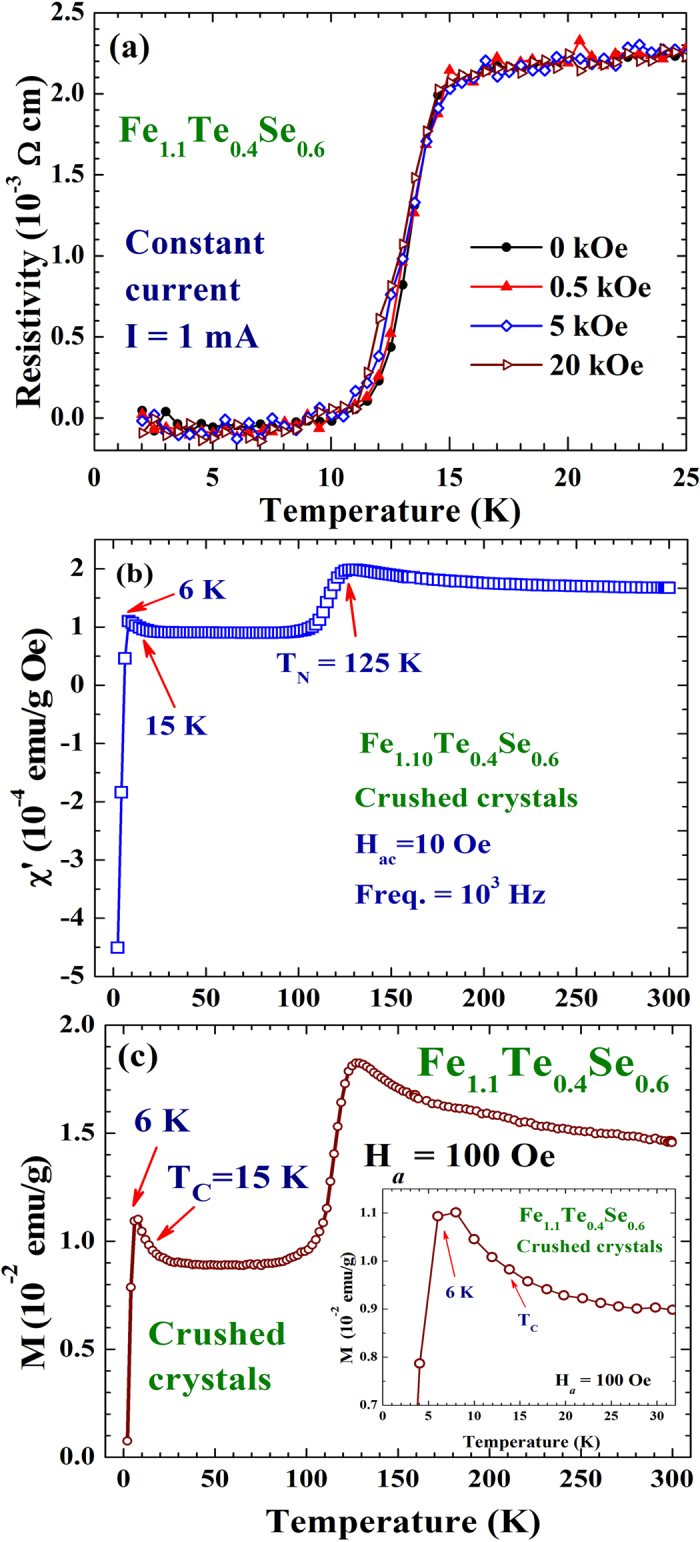
(**a**) Temperature dependencies of the electrical resistivity of the x = 0.6 sample, measured at various applied magnetic fields, employing the constant current mode of I = 1 mA. (**b**) χ′(T) curve of the x = 0.6 compound, measured using a probing field with a root-mean-square strength of 10 Oe and a frequency of 10^3^ Hz. (**c**) M(T) curve of the x = 0.6 compound, measured at H_*a*_ = 100 Oe. The inset shows the M(T) curve in the critical regime using an expanded scale.

**Figure 4:**
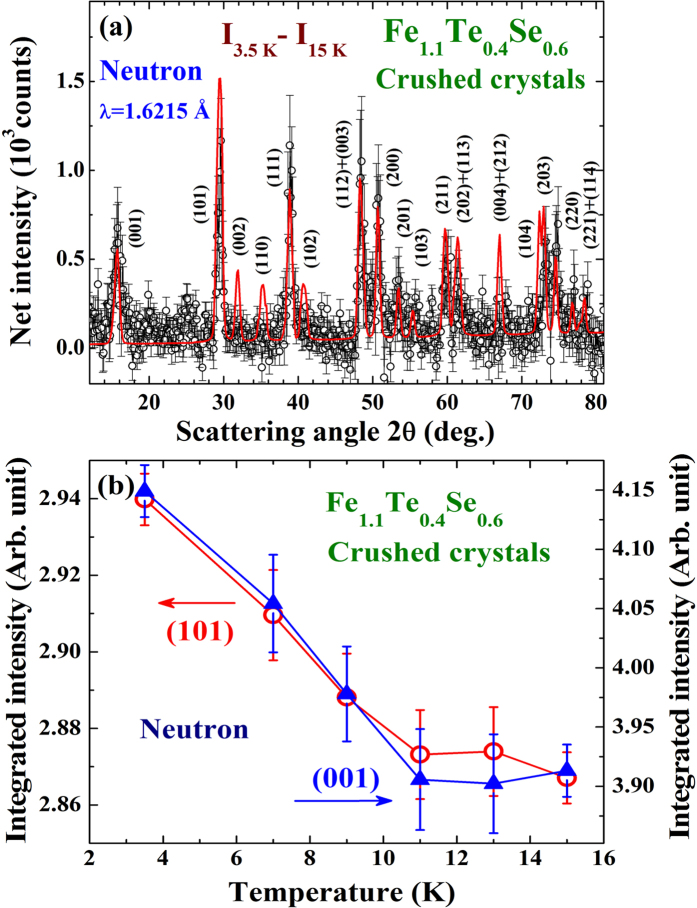
(**a**) Magnetic diffraction pattern of the x = 0.6 sample observed at 3.5 K, where the diffraction pattern at 15 K that served as the non-magnetic background has been subtracted. (**b**) Temperature dependencies of the integrated intensities of the (001) (filled triangles) and (101) (open circles) reflections of the x = 0.6 sample.

**Figure 5:**
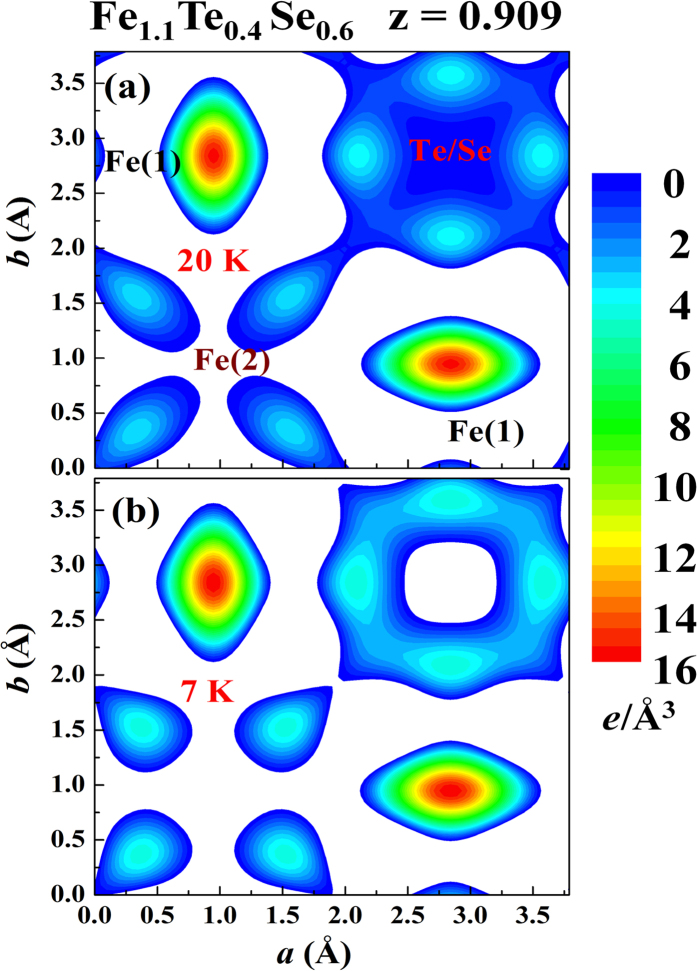
Electronic charge densities in the z = 0.909 crystallographic plane of the x = 0.6 sample at (**a**) 20 K and (**b**) 7 K, as inferred from the x-ray diffraction data. The color bars are in units of *e*/Å^3^.

Superconductivity with T_C _= 15 K is seen in the resistivity curve of the x = 0.6 compound and T_C_ remains essentially unaffected at H_*a *_= 20 kOe (Fig. 3a). χ′(T) and M(T) reveal a T_N_ = 125 K (Fig. 3b and Fig. 3c). SDS is see below 6 K in both the χ′(T) and M(T) curves, with small but clearly visible increases of χ′ and M are again seen upon cooling through the superconducting transition before SDS becomes dominating (Fig. 3b and inset to Fig. 3c). The increases in M and χ′ upon cooling through T_C_ = 15 K (revealed in the resistivity) signal that there is a FM component developed through the transition, with a strength that is larger than the SDS could screen. The reductions in M and χ′ upon cooling to below 6 K indicate the temperature below which SDS becomes dominating, rather than the T_C_ of the compound. Direct comparisons of the isothermal magnetization M(H_*a*_) curves taken below and above T_C_ of the two samples can be found in the supplementary material. Neutron powder diffraction was employed with the powdered sample (crushed from crystals) to reveal the overall magnetic intensities that developed through the superconducting transition. The difference pattern between the diffraction patterns taken at 3.5 and 15 K reveals a series of peaks at the FM positions, but none at the AFM positions, that developed through the superconducting transition (Fig. 4a). The technique of taking the difference between the patterns collected at 3.5 and 15 K is applicable, since the changes in the scattering angles from the thermal contraction [by 0.013^o^ in (111) and 0.026^o^ in (113) reflections] are smaller than the instrumental resolution (with a full width at half maximum of 0.63^o^ for the peak at scattering angle of 40^o^) and measuring steps of 0.125^o^. Direct comparisons of the neutron diffraction patterns taken at 3.5 and 15 K of the x = 0.6 sample and taken at 6 and 18 K of the x = 0.4 sample can be found in the supplementary material. The magnetic diffraction pattern was analyzed using the General Structure Analysis System (GSAS) program, resulting a magnetic moment for the Fe(2) ion that is indistinguishable from that of the Fe(1) ion. The size of the moment was then obtained by comparing the magnetic intensities to the nuclear intensity of the (101) reflection, assuming the same moments for the Fe(1) ion and for the Fe(2) ion. The magnetic diffraction pattern can be described (solid curves in Fig. 4a) by having FM moments of 0.35 μ_B_, pointing along the crystallographic *c*-axis direction, for the Fe ions at the lattice and the interstitial sites. The additional FM component is revealed below 11 K (Fig. 4b). Upon cooling from 20 to 7 K (through the superconducting transition), the outer electronic charge densities of Fe(2) become less intense and less connected with the Te/Se ions, while that of Te/Se become more intense and more extended (Fig. 5), indicating that the cooling causes a flow of electronic charges from the Fe(2) ions to the Te/Se sites.

The effects of excess iron on electronic charge redistribution and enhanced magnetization in Fe_1+*y*_(Te_1-*x*_Se_*x*_) can be further confirmed via density functional theory (DFT) calculations. To make sure that all Fe(1) atoms experience the same Te/Se crystal field in our DFT calculation, we start with a 16-atom FeTe_0.5_Se_0.5_ supercell, where Se and Te are placed in the same layer in a check-board manner, namely, all Se atoms are neighbored by Te atoms, and vice versa. Adding an interstitial Fe(2) to such a supercell forms Fe_1.125_Se_0.5_Te_0.5_, a reasonable approximation to the experiments. The effect of temperature is modeled by adopting structural parameters (lattice constants and atomic positions) obtained from high-resolution neutron diffraction measurements at designated temperatures. In this sense the effects from charge redistribution that modifies lattice constants and atomic positions are included in the consideration, but the effects from electron paining are neglected. Our DFT calculations were performed using the Quantum ESPRESSO code^18^ and GBRV high-throughput pseudopotentials (a set of ultra-soft pseudopotentials generated with Vanderbilt method)^19^. Given the weaker electron correlation (in contrast to cooperates and Mott insulators) and the metallic nature of iron chalcogenide, electronic structures of this class of materials predicted by standard DFT functional agree better with experimental results^20^,^21^,^22^. In our calculation, the PBE-type generalized gradient approximation (GGA)^23^ is adopted.

**Figure 6:**
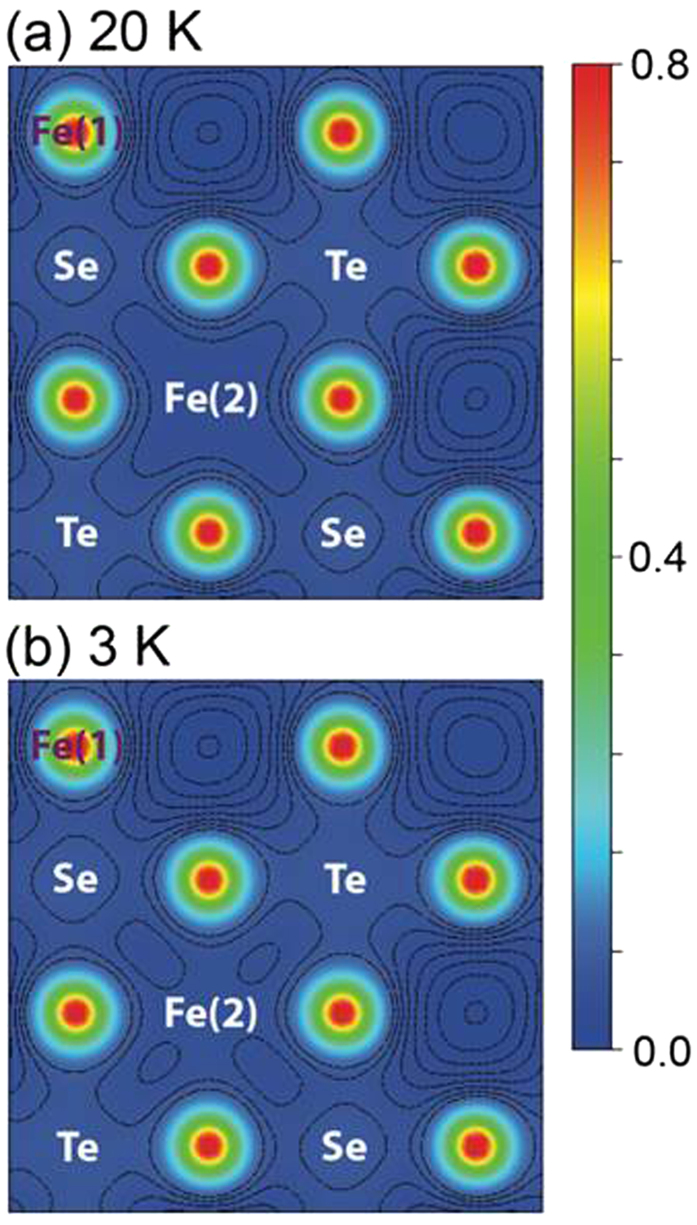
Calculated electron density of Fe_1.125_Te_0.5_Se_0.5_ on the z = 0.909 crystallographic plan at (**a**) 20 K and (**b**) 3 K. The color bar is in the unit of *e*/a.u.^2^, and the projection of each atomic site on this plan is indicated. The contour lines, plotted in log scale, indicate the region where the electronic charge density is lower than 0.1*e*/a.u.^2^.

The calculated electron density of Fe_1.125_Te_0.5_Se_0.5_ on the *z* = 0.909 crystallographic plan (in between Fe(1) and Fe(2)/Te-Se layer) using the 20 and 3 K structural parameters are shown in Fig. 6, where the color bar is in the unit of *e*/a.u.^2^, and the projection of each atomic site on this plan is indicated. The structural parameters used in the calculation (listed in the supplementary material) were obtained on two powder samples of Fe(Te_0.5_Se_0.5_) and Fe_1.125_(Te_0.5_Se_0.5_) determined by high-resolution neutron diffraction measurements. Details of the sample fabrication and characterization can be found in the supplementary material. To better visualize the electron charge distribution in the region with low density (<0.1 *e*/a.u.^2^), contour lines in log scale are plotted as well. The most remarkable difference caused by the temperature change is the electron density around the Fe(2) site. At 20 K (Fig. 6a), the contour of electron density around the Fe(2) site exhibits an extended character in between the Fe(2) and Se/Te sites. In contrast, the contours of electron density obtained at 3 K (Fig. 6b) exhibit a more isolated-island type of structure. This result is consistent with the electron density observed in diffraction experiments (Fig. 5), strongly suggesting the redistribution of electronic charge from the Fe(2) toward the Te/Se sites through the superconducting transition.

The effect of excess iron on the magnetization of Fe(Te,Se) can be examined by comparing the overall magnetization per supercell and the magnetic moment of each type of iron in Fe_1.125_Te_0.5_Se_0.5_ and FeTe_0.5_Se_0.5_. The results of the calculations using the structural parameters obtained at 20 K (normal state) and 3 K (superconducting state) are summarized in Table 1. It should be pointed out that the total magnetization per cell is slightly smaller than the sum of the magnetic moment of each type of iron, as small but negative magnetic moments are generated for the Se and Te ions as well. As shown in Table 1, the excess Fe(2) irons, enhance the overall magnetization by its own significantly larger magnetic moment and its effect on nearby Fe(1) ions. At 20 K and 3 K, the presence of Fe(2) ions results in an increase of the total magnetization of a supercell by 2.85 and 3.22 μ_B_, respectively. These increases are significantly larger than the average spin moment of Fe(1) ion in the parent compound (2.21 μ_B_ at 20 K and 2.10 μ_B_ at 3 K), where the average spin moment is defined as the total magnetization divided by the number of Fe(1) atoms. This results can be understood by the localized iron spin moment listed alongside: the Fe(2) ion has the largest moment; it enhances the magnetic moment of the nearby Fe(1) ions, but barely affects the distant ones. Evidently from our calculations, the presence of Fe(2) ions affects the magnetization of Fe(Te,Se) more at 3 K than at 20 K, suggesting that the excess iron plays a more significant role affecting the magnetic property in the superconducting state than in the normal state.

**Figure 7:**
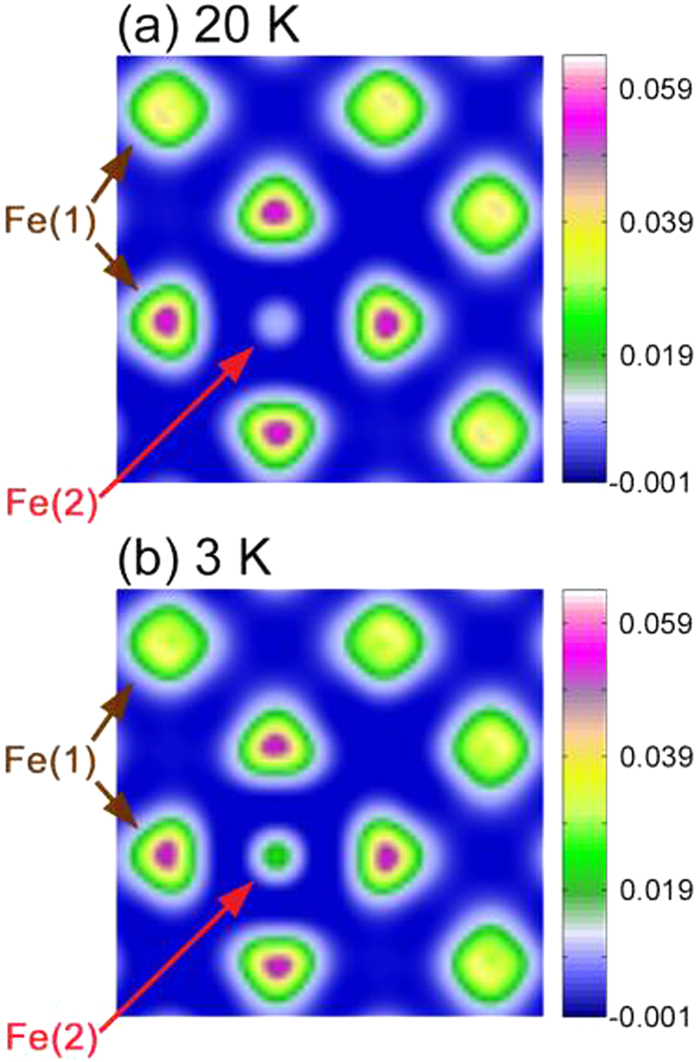
Contour plots of the spin density, s(r), in the (0 0 0.89) plane of Fe_1.125_Te_0.5_Se_0.5_ at (**a**) 20 K and (**b**) 3 K. The spin density around the Fe(2) ions is noticeably higher at 3 K than that at 20 K.

The effect of excess iron can be visualized by plotting the spin density contour map *s*(**r**) ≡ *ρ*_↑_(**r**) – *ρ*_↓_(**r**) as well, where *ρ*_↑_(**r**) and *ρ*_↓_(**r**) indicate the spin-up and spin-down electron density distributions, respectively. Figure 7a,b show the contour maps of *s*(**r**) in the (0 0 0.89) plane, a horizontal plane between the Fe(2) and the Fe(1) layers, at 20 and 3 K, respectively. Remarkably, in addition to magnetic spin density does appear in the Fe(2) ions, the distribution of the spin density of the nearby Fe(1) ion is largely different from that of the distanced Fe(1) ions. The Fe(2) ions distort the spin density of the nearby Fe(1) ions from having a more-or-less rounded distribution to a triangular one. Comparing the two structures, a higher spin density around the Fe(2) ions and less rounded spin density distribution around the nearby Fe(1) ions can be observed at 3 K.

## Conclusions

In this study, the development of an additional ferromagnetic component in the superconducting state is identified, which links directly to the occurrence of electronic charge redistribution, in which a small portion of the electronic charges around the Fe(1) and Fe(2) sites flow to the Te/Se sites on cooling through the superconducting transition. In the compounds with excess Fe ions, the divalent Te/Se bonds are shared among the Fe(1) ions on the lattice sites and the Fe(2) ions on the interstitial sites. Some of the 4*s* electrons of the Fe(1) ions will be left unbounded to form monovalent Fe(1)^1+^, while the majority of the Fe(1) ions are divalent Fe(1)^2+^. The observation of a small portion of the electronic charges around the Fe(2) sites flowing to the Te/Se sites on cooling through the superconducting transition shows that some of the Fe(2) ions are indeed divalent at low temperatures. The flow of electronic charges from the Fe(1) and Fe(2) sites into the Te/Se sites results in an increase of the number of divalent Te^2−^/Se^2−^ ions upon cooling through the superconducting transition, which in average gives rise to a larger Te^2−^/Se^2−^ ion and drives the lattice to expand. Although the alternation in the Fe ionic size may not be noticeable upon losing 4*s* electrons from the Fe^1+^ ions, it does cause an increase of the number of divalent Fe^2+^ ions on the account of a decrease of the number of monovalent Fe^1+^ ions. This situation occurs on the lattice Fe(1) sites as well as on the interstitial Fe(2) sites. The Fe(1) and Fe(2) spins become ordered into an antiferromagnetic spin arrangement at a temperature which is much higher than T_C_. Changes of the numbers of monovalent and divalent Fe ions on the Fe(1) and Fe(2) sites could alter the overall magnetic interactions. It is the changes in the locations of monovalent and divalent Fe ions, triggered by electronic charge redistribution through superconducting transition, which gives rise to the evolution of magnetic structure from its high-temperature phase in the normal state to the low-temperature phase in the superconducting state. Apparently, thermal loosening of the superconducting pairs allows the electrons to flow from the superconducting layers to the interstitial Fe sites, which alters the magnetic structure of the compound. We remark that the results of this study are concluded from Te/Se = 60/40 and 40/60 compounds. Although it means to suggest that similar effects might appear in the Te/Se = 50/50 compounds, but investigations of 50/50 systems are needed to confirm or disapprove the suggestion. Current study has demonstrated that a larger amount of electronic charge flow will appear in the compound with more excess Fe ions, but the effects from the Te/Se composition ratio are still to be investigated further. Studies with compounds of various Te/Se compositions might help to clear the physical picture of electronic charge redistribution built in the present study. Finally, the changes in the valences of Fe(1) and Fe(2) ions through the superconducting transition could also be revealed in Mossbauer spectroscopy.

## Additional Information

**How to cite this article**: Li, W.-H. *et al.* Development of a ferromagnetic component in the superconducting state of Fe-excess Fe_1.12_Te_1-x_Se_x_ by electronic charge redistribution. *Sci. Rep.* **5**, 10951; doi: 10.1038/srep10951 (2015).

## Supplementary Material

Supplementary Information

## Figures and Tables

**Table 1:** Calculated total magnetization and the magnetic moments of the Fe(1) and Fe(2) ions in Fe_1.125_Te_0.5_Se_0.5_ (16-atom supercell FeTe_0.5_Se_0.5_) at 20 and 3 K.

	**Total magnetization (μ_B_ per supercell)**	**Magnetic moment of iron (μ_B_)**		
		**Fe(2)**	**Fe(1) nearby Fe(2)**	**Fe(1)**
20 K structure with Fe(2)	20.53	2.60	2.35	2.31
20 K structure without Fe(2)	17.68	–	–	2.25
3 K structure with Fe(2)	19.99	2.61	2.29	2.24
3 K structure without Fe(2)	16.77	–	–	2.20
