# Supplementary material for: Development of a ferromagnetic component in the superconducting state of Fe-excess Fe1.12Te1-xSex by electronic charge redistribution
Source: Sci Rep. 2015 Jun 16;5:10951. doi: 10.1038/srep10951 (PMC5155544; doi:10.1038/srep10951)
Supplement: Supplementary Information [file srep10951-s1.pdf]

Supplementary Information

Development of a ferromagnetic component in the superconducting state of Fe-excess $Fe_{1.12}Te_{1-x}Se_x$ by electronic charge redistribution

Wen-Hsien Li[1], Sunil K. Karna[1], Han Hsu[1], Chi-Yen Li[1], Chi-Hung Lee[1], Raman Sankar[2], and Fang Cheng Chou[2]
[1]Department of Physics, National Central University, Jhongli 32001, Taiwan
[2]Center for Condensed Matter Sciences, National Taiwan University, Taipei 10617, Taiwan

Sample fabrication. Stoichiometric amounts and well mixed powders of Fe, Se and Te (99.999% pure) were pelletized and sealed in evacuated (10^{-4} torr) quartz tubes. The sealed quartz tubes containing the pellets were heated at 800 °C for 10 hours. This was followed by natural furnace-cooling to room temperature. The sintered pellets were again ground, pelletized and sealed, before being heated at 910 °C for 30 hours. The temperature was then gradually ramped down to room temperature over 40 hours.

Instrumentation. The x-ray diffraction measurements were performed on a Bruker D8 ADVANCE diffractometer, employing the standard setup. The neutron diffraction measurements were conducted at the Bragg Institute, ANSTO, using the high-resolution powder diffractometer Echidna [λ = 1.6215 Å defined by Ge (335) crystals] for crystalline structure analysis, and the thermal triple-axis spectrometer Taipan [λ = 2.35 Å defined by PG(002) crystals at both the monochromator and analyzer positions] for single crystal diffraction. For the powder diffraction measurements, ~7 g of the sample was loaded into a cylindrical vanadium-can which gave rise to no measureable neutron diffraction peak. The sample temperature was controlled using a He-gas closed-cycle refrigerator system. Chemical analysis by means of energy dispersive x-ray spectroscopy (EDXS) was also performed to characterize the elemental compositions of the samples. The EDXS spectra were taken with a HORIBA EX-220 detector attached to a HITACHI S-4200 scanning electron microscope, employing a standard setup. The magnetization, ac magnetic susceptibility and resistivity measurements were performed on a Physical Property Measurement System (PPMS), employing the standard setups.

Sample characterization. X-ray and neutron diffractions were used to characterize the samples. No obvious differences were found in the x-ray diffraction patterns taken from different portions of each powdered sample. The high-resolution neutron diffraction patterns were analyzed using the General Structure Analysis System (GSAS) program[S1] following the Rietveld profile refining method. The observed and calculated (assuming a *P*4/*nmm* symmetry) neutron diffraction patterns of the six samples are shown in Figs. S1(a)~S1(f), with their differences plotted at the bottom. They agree very well. The refined

structural parameters are listed in Tables S1a~S1f. All sites are fully occupied with a 10% excess of Fe ions which appears at the interstitial sites, giving rise to a chemical composition of $Fe_{1.10}Se_{0.4}Te_{0.6}$ for the x = 0.6 polycrystalline sample. Elemental analysis using EDXS images taken from 18 different portions of the sample gives an atomic ratio of Fe:Se:Te = 52.6(5):19.1(6):28.4(4), which agrees well with the composition obtained from the neutron diffraction analysis. The chemical compositions obtained were $Fe_{1.12}Te_{0.6}Se_{0.4}$ for the x = 0.4 sample, $Fe_{1.03}Te_{0.61}Se_{0.39}$ for the y = 0.03 sample, $Fe_{1.08}Te_{0.61}Se_{0.39}$ for the y = 0.08 sample, $FeTe_{0.51}Se_{0.49}$ and $Fe_{1.125}Te_{0.51}Se_{0.49}$ for the two x = 0.5 samples. There is no structural change or lattice distortion that can be identified from the high-resolution neutron diffraction patterns for all samples between 2 and 300 K. The structural parameters, which were used in DFT calculation, obtained on two x = 0.5 powder samples at 3 and 20 K are listed in Table S2.

Magnetization. The isofield $\chi'(T)$ and M(T) curves of the x = 0.4 sample, with a temperature range covering 2 to 300 K, are shown in Figs. S2a and S2b, respectively. The anomaly in $\chi'(T)$ at 130 K for the antiferromagnetic (AFM) ordering of the Fe ions is smeared by an applied magnetic field of H_a = 10 kOe. The AFM transition, nevertheless, is clearly revealed in the M(T) curve taken at 10 kOe. The isothermal magnetization $M(H_a)$ curves of the x = 0.4 and 0.5 samples, measured above and below T_C, are shown in Figs. S3(a) and S3(b), respectively, revealing that the magnetizations measured below T_C are noticeably larger than the corresponding values measured above T_C at all fields studied.

Magnetic neutron diffraction pattern. The neutron diffraction patterns taken at 3.5 and 15 K of the x = 0.6 sample are displayed together in Fig. S4 for a direct comparison. These patterns contain diffraction intensities from nuclear reflections as well as from magnetic reflections. Clearly, the intensities at the positions of nuclear reflections of the 3.5 K pattern are noticeably higher, indicating the development of additional ferromagnetic intensities upon cooling through the superconducting transition. Similar behavior was also observed in the x = 0.4 sample, as shown in Fig. S5.

Additional comment. The increases in magnetization upon entering the superconducting transition on cooling are the most direct evidences for that the superconductivity and ferromagnetism are occurring together. This piece of information was obtained on a single crystal, where no twining was found but generated nice Gaussian neutron rocking curves. It is very unlikely that superconductivity and ferromagnetism are generated from different regions of the sample.

Reference

S1. Larson, A. C. & von Dreele, R. B., General Structure Analysis System Report LA-UR-86-748 (Los Alamos, NM: Los Alamos National Laboratory) (2004).

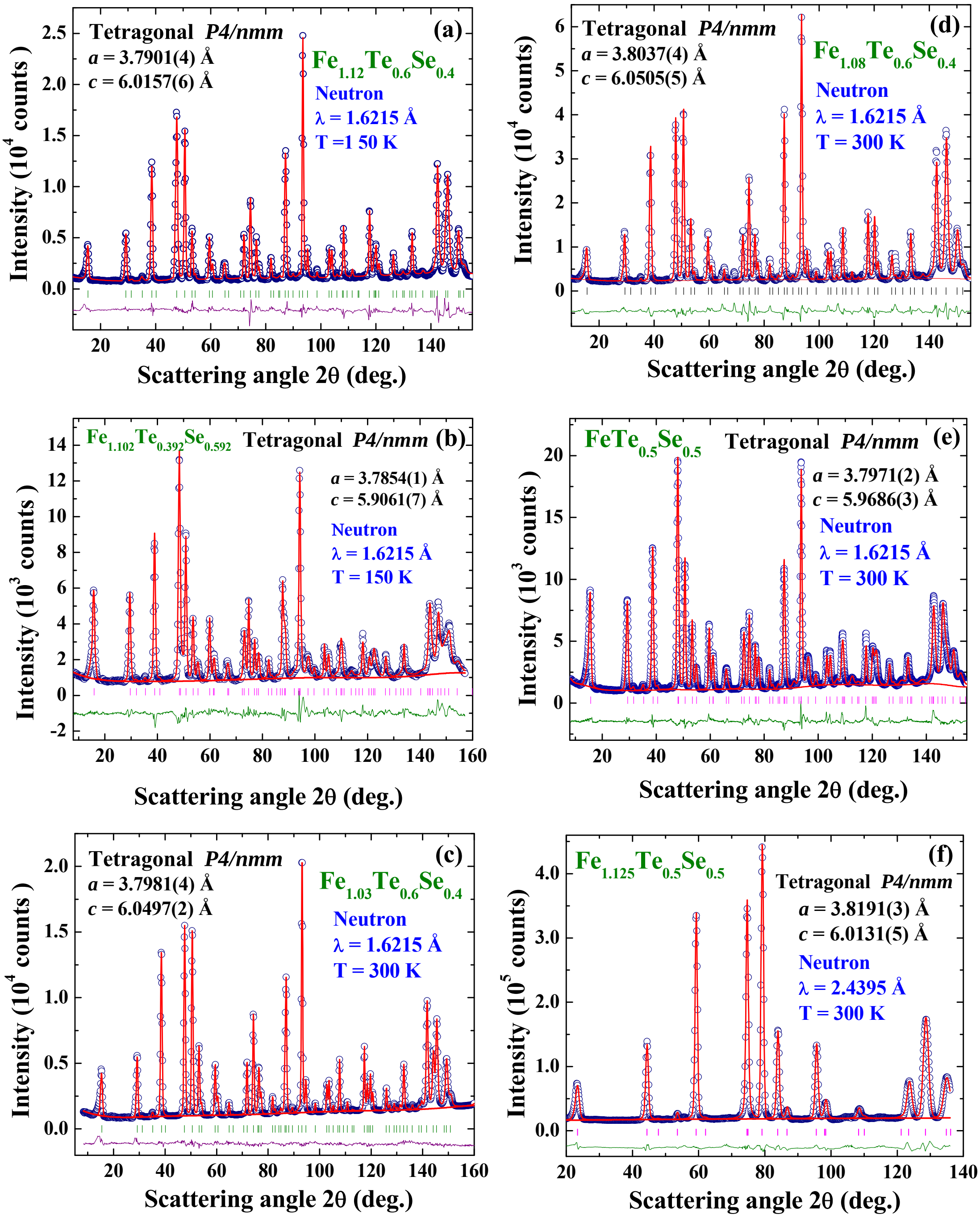

Figure S1: Observed (circles) and fitted (solid lines) high-resolution neutron powder diffraction patterns of the (a) x = 0.4, (b) x = 0.6, (c) y = 0.03, (d) y =0.08, and (e~f) x =0.5 compounds, assuming a tetragonal symmetry of the space group *P4/nmm*. The differences between the calculated and observed patterns are plotted at the bottom. The solid vertical lines mark the calculated positions of the Bragg reflections of the proposed crystalline structure.

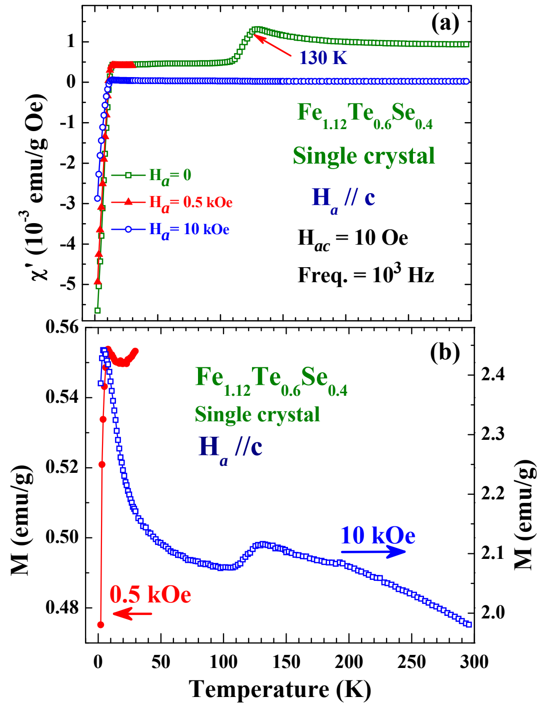

Figure S2: (a) χ'(T) curves of the x = 0.4 crystal, measured at H_a = 0 (open squares), 0.5 kOe (filled triangles) and 10 kOe (open circles). (b) M(T) curves of the x = 0.4 crystal, measured at H_a = 0.5 kOe (filled circles) and 10 kOe (open squares).

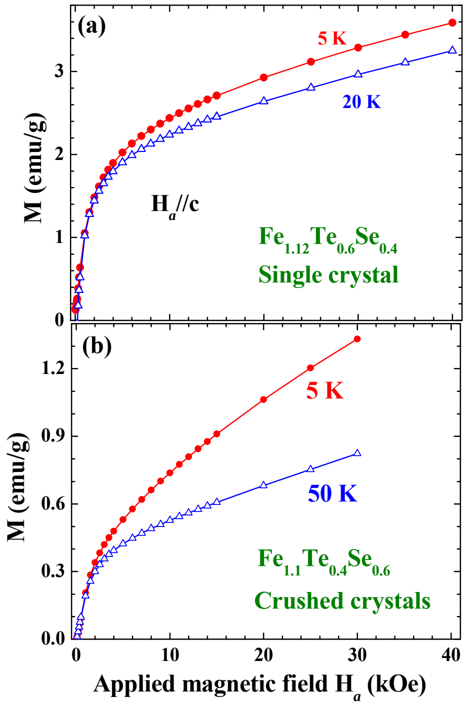

Figure S3: M(H_a) curves of the (a) x = 0.4 and (b) x = 0.6 compounds, measured below and above the superconducting transition.

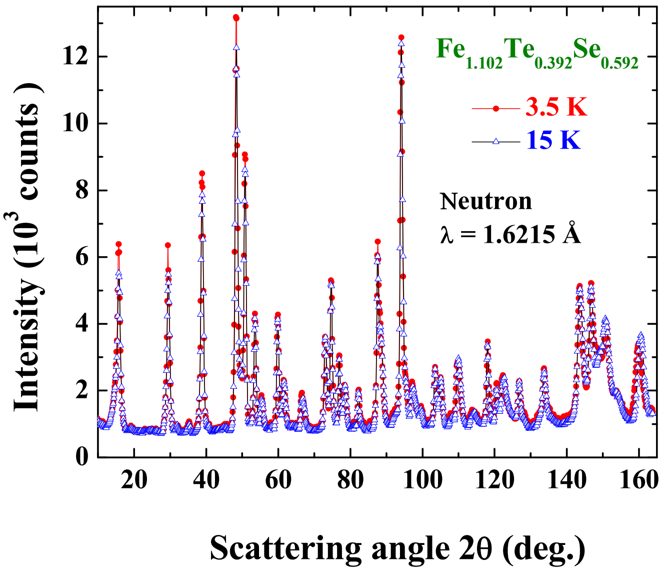

Figure S4: Direct comparisons of the neutron diffraction patterns taken at 3.5 K (filled circles) and 15 K (open triangles) of the x = 0.6 compound.

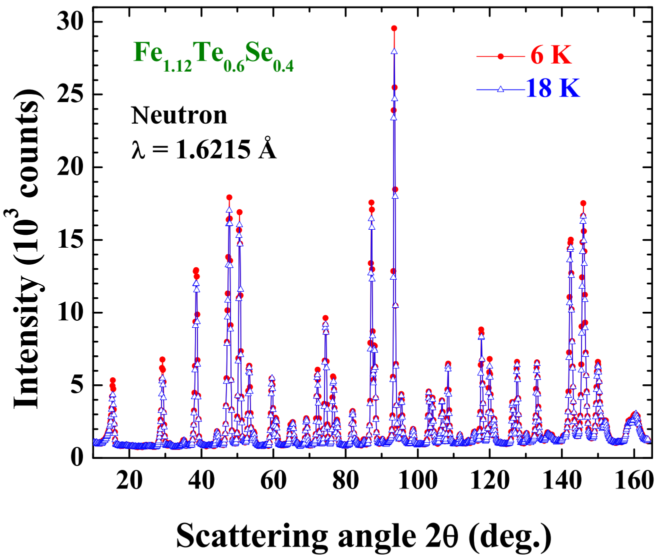

Figure S5: Direct comparisons of the neutron diffraction patterns taken at 6 K (filled circles) and 18 K (open triangles) of the x = 0.4 compound.

Table S1: Lists of refined structural parameters of (a) $Fe_{1.12}Te_{0.61}Se_{0.39}$ at 150 K, (b) $Fe_{1.10}Te_{0.39}Se_{0.59}$ at 150 K, (c) $Fe_{1.03}Te_{0.61}Se_{0.39}$ at 300 K, (d) $Fe_{1.08}Te_{0.61}Se_{0.39}$ at 300 K, (e) $FeTe_{0.51}Se_{0.49}$ at 300 K, and (f) $Fe_{1.125}Te_{0.51}Se_{0.49}$ at 300 K. B_{iso} represents the isotropic temperature parameter.

(a) Tetragonal *P4/nmm* (No. 129, Z = 2), T = 150 K, *a* = *b* = 3.7901(4) Å, *c* = 6.0157(6) Å

Atom	x	y	z	Site	B_{iso} (Å^2)	Occupancy
Fe(1)	0.75	0.25	0	2*a*	0.64(4)	1
Te	0.25	0.25	0.2791(3)	2*c*	1.09(7)	0.61(2)
Se	0.25	0.25	0.2791(3)	2*c*	1.09(7)	0.39(2)
Fe(2)	0.25	0.25	0.7388(4)	2*c*	2.07(5)	0.123(4)

$\chi^2 = 2.915$, $R_p = 3.89\%$, $R_{wp} = 4.56\%$

(b) Tetragonal *P4/nmm* (No. 129, Z = 2), T = 150 K, *a* = *b* = 3.7854(1) Å, *c* = 5.9061(7)Å

Atom	x	y	z	Site	B_{iso} (Å^2)	Occupancy
Fe(1)	0.75	0.25	0	2*a*	0.89(4)	1
Te	0.25	0.25	0.2671(5)	2*c*	0.99(7)	0.392(4)
Se	0.25	0.25	0.2671(5)	2*c*	0.99(7)	0.592(4)
Fe(2)	0.25	0.25	0.7366(3)	2*c*	3.01(8)	0.102(2)

$\chi^2 = 2.933$, $R_p = 3.75\%$, $R_{wp} = 4.65\%$

(c) Tetragonal *P4/nmm* (No. 129, Z = 2), T = 300 K, *a* = *b* = 3.7981 (4) Å, *c* = 6.0497(2) Å

Atom	x	y	z	Site	B_{iso} (Å^2)	Occupancy
Fe(1)	0.75	0.25	0	2*a*	0.77(2)	1
Te	0.25	0.25	0.2711(3)	2*c*	1.30(3)	0.608(2)
Se	0.25	0.25	0.2711(3)	2*c*	1.30(3)	0.394(2)
Fe(2)	0.25	0.25	0.7080(6)	2*c*	2.29(4)	0.032(3)

$\chi^2 = 2.014$, $R_p = 3.12\%$, $R_{wp} = 3.84\%$

(d) Tetragonal *P4/nmm* (No. 129, Z = 2), T = 300 K, *a* = *b* = 3.8037(4) Å, *c* = 6.0505(5) Å

Atom	x	y	z	Site	B_{iso} (Å^2)	Occupancy
Fe(1)	0.75	0.25	0	2*a*	0.84(3)	1
Te	0.25	0.25	0.2882(5)	2*c*	1.22(4)	0.608(2)
Se	0.25	0.25	0.2882(5)	2*c*	1.22(4)	0.392(2)
Fe(2)	0.25	0.25	0.7313(6)	2*c*	1.66(5)	0.084(4)

$\chi^2 = 2.513$, $R_p = 3.15\%$, $R_{wp} = 4.27\%$

(e) Tetragonal *P4/nmm* (No. 129, Z = 2), T = 300 K, $a = b = 3.7971(4)$ Å, $c = 5.9686(3)$ Å

Atom	x	y	z	Site	B_{iso}($Å^2$)	Occupancy
Fe(1)	0.75	0.25	0	2*a*	0.75(5)	1.003(2)
Te	0.25	0.25	0.2675(3)	2*c*	1.06(3)	0.514(2)
Se	0.25	0.25	0.2675(3)	2*c*	1.06(3)	0.493(2)

$\chi^2 = 1.447$, $R_p = 3.08\%$, $R_{wp} = 4.04\%$

(f) Tetragonal *P4/nmm* (No. 129, Z = 2), T = 300 K, $a = b = 3.8191(3)$ Å, $c = 6.0131(5)$ Å

Atom	x	y	z	Site	B_{iso}($Å^2$)	Occupancy
Fe(1)	0.75	0.25	0	2*a*	0.95(5)	1.002(6)
Te	0.25	0.25	0.2684(3)	2*c*	1.05(6)	0.514(2)
Se	0.25	0.25	0.2684(3)	2*c*	1.05(6)	0.493(2)
Fe(2)	0.25	0.25	0.7126(5)	2*c*	1.57(4)	0.125(4)

$\chi^2 = 3.514$, $R_p = 4.52\%$, $R_{wp} = 5.25\%$

Table S2: Lists of refined structural parameters of (a) $FeSe_{0.51}Te_{0.49}$ and (b) $Fe_{1.125}Se_{0.51}Te_{0.49}$ at 3 and 20 K.

(a) Tetragonal *P4/nmm* (No. 129, Z = 2), T = 3 K, *a* = *b* = 3.7948(3) Å, *c* = 5.9339(5) Å.

Atom	X	y	z	Site	B_{iso}($Å^2$)	Occupancy
Fe	0.75	0.25	0	2*a*	0.58(4)	1.00
Te	0.25	0.25	0.2656(2)	2*c*	0.78(5)	0.514(2)
Se	0.25	0.25	0.2656(2)	2*c*	0.78(5)	0.493(2)

χ^2 = 2.864, R_p= 3.32%, R_{wp}= 4.18%

T = 20 K, *a* = *b* = 3.7943(3) Å, *c* = 5.9336(5) Å

Atom	X	y	z	Site	B_{iso}($Å^2$)	Occupancy
Fe(1)	0.75	0.25	0	2*a*	0.68(5)	1
Te	0.25	0.25	0.2679(3)	2*c*	0.82(6)	0.514(2)
Se	0.25	0.25	0.2679(3)	2*c*	0.82(6)	0.493(2)

χ^2 = 2.514, R_p= 3.12%, R_{wp}= 4.05%

(b) Tetragonal *P4/nmm* (No. 129, Z = 2), T = 3 K, *a* = *b* = 3.8241(3) Å, *c* = 5.9874(5) Å.

Atom	X	y	z	Site	B_{iso}($Å^2$)	Occupancy
Fe(1)	0.75	0.25	0	2*a*	0.79(4)	1.00
Te	0.25	0.25	0.2684(2)	2*c*	0.92(5)	0.514(2)
Se	0.25	0.25	0.2684(2)	2*c*	0.92(5)	0.493(2)
Fe(2)	0.25	0.25	0.7074(6)	2*c*	1.15(4)	0.125(4)

χ^2 = 3.174, R_p= 3.82%, R_{wp}= 4.29%

T = 20 K, *a* = *b* = 3.8297(3) Å, *c* = 6.0170(5) Å.

Atom	x	y	z	Site	B_{iso}($Å^2$)	Occupancy
Fe(1)	0.75	0.25	0	2*a*	0.82(5)	1
Te	0.25	0.25	0.2682(3)	2*c*	0.95(6)	0.514(2)
Se	0.25	0.25	0.2682(3)	2*c*	0.95(6)	0.493(2)
Fe(2)	0.25	0.25	0.7129(5)	2*c*	1.27(4)	0.125(4)

χ^2 = 3.213, R_p= 3.93%, R_{wp}= 4.58%
